# Targeting neuroinflammation - a potential for anti-aging interventions

**DOI:** 10.18632/aging.101296

**Published:** 2017-09-22

**Authors:** Gillian Cady, Marianna Sadagurski

**Affiliations:** Department of Biological Sciences, Integrative Biosciences Center, Wayne State University, Detroit, MI 48202, USA

**Keywords:** hypothalamic inflammation, anti-aging drugs, sex differences, longevity

Increased inflammatory activity and associated upregulation of inflammatory cytokines are responsible for normal brain aging and local glial cell activation. The hypothalamus is the main controller of various physiological processes, including energy regulation and metabolism [1]. Recent research in the cross-disciplinary field of neurobiology and aging revealed a fundamental role of age-associated hypothalamic inflammation as the essential process involved in neural mechanisms of aging. Slowing of the murine aging process can be achieved by inhibiting activation of hypothalamic proinflammatory axis comprising IκB kinase-β (IKKβ) and its downstream nuclear transcription factor NF-κB (IKKβ/NF-κB signaling) [1]. Our recent studies demonstrated that age-associated hypothalamic inflammation is reduced in the long-lived Snell dwarf, Ames dwarf or growth hormone receptor deficient (GHRKO) mice [2]. Thus, a reduction in hypothalamic –mediated inflammation may attenuate the aging process as well as mitigate age-associated disorders.

Recent NIA Interventions Testing Program (ITP) lifespan results demonstrated significant gender differences of pharmacological and dietary inter-ventions. Pharmacological treatments, including aspirin, nordihydroguaiaretic acid (NDGA), acarbose (ACA), Protandim, and 17-α estradiol (17αE2), extend mouse lifespan to a greater degree in males [3, 4]. By contrast, rapamycin, an inhibitor of the mTOR pathway, extends the median and maximal lifespan of both male and female mice [5]. Similarly, caloric restriction (CR) markedly increases both overall mean and maximum life span among different mouse stocks [6]. In our new study, we demonstrated that age-associated hypo-thalamic inflammation is similarly reduced solely in males at 12 months of age by ACA and 17αE2, and at 22 months of age in NDGA-treated mice. This effect was not observed either in drug-treated female mice or in the hippocampus of the drug-treated animals. However, CR significantly reduced hypothalamic inflammation in both genders at 12-months of age [7]. This lends credence to the concept that inhibition of the hypothalamic inflammatory responses may result in significant gains in the murine lifespan. Moreover, our data provide evidence that drugs that extend lifespan might be effective in inhibiting hypothalamic inflam-matory processes in a gender-dependent manner.

The activity of ACA, NDGA, and 17αE2 as anti-inflammatory agents opens new avenues for mechanistic testing and may prove valuable for assessing gender differences in a murine model as well as attenuating the effects of aging and associated neurodegenerative diseases. The underlying causes for gender differences are not fully understood. It was recently hypothesized that male lifespan extension with ACA and 17aE2 are due to improved glucose homeostasis [8]. Indeed, ACA and 17aE2, which lead to mouse lifespan extension principally in males, also produce male-specific improvements in glucose tolerance and elevations in hepatic mTORC2 activity [8]. Furthermore, gender related responses to these drugs are influenced by both male and female gonadal hormones, suggesting that castrated males would show little to no lifespan benefit from either drug whereas ovariectomy might allow females to benefit from one or both of these interventions. While this work does not establish whether the beneficial effects of ACA and 17aE2 on age-associated hypothalamic inflammation is also affected by gonadal hormones, it is reasonable to hypothesize that hypothalamic areas that regulate gender and sexual behavior are modified by the anti-aging drugs.

Evidence that multiple models of delayed aging have commonality may have a major impact on bio-gerontology. Attention to processes such as hypothalamic inflammatory responses, may lead to ate-nuation of the aging process. A deeper understanding of the physiological and hormonal causes for gender discrepancies in lifespan extension with anti-aging drugs, and for age-associated hypothalamic inflam-mation could provide significant insights into mechanisms underlying the aging process and guide development of novel therapeutic agents for both genders.
